# Development of a novel nanoemulgel formulation containing cumin essential oil as skin permeation enhancer

**DOI:** 10.1007/s13346-021-01025-1

**Published:** 2021-07-17

**Authors:** Katayoun Morteza-Semnani, Majid Saeedi, Jafar Akbari, Mohammad Eghbali, Amirhossein Babaei, Seyyed Mohammad Hassan Hashemi, Ali Nokhodchi

**Affiliations:** 1 Department of Medicinal Chemistry, Faculty of Pharmacy, Mazandaran University of Medical Sciences, Sari, Iran; 2 Pharmaceutical Sciences Research Center, Hemoglobinopathy Institute, Mazandaran University of Medical Sciences, Sari, Iran; 3 Department of Pharmaceutics, Faculty of Pharmacy, Mazandaran University of Medical Sciences, Sari, Iran; 4 Student Research Committee, Faculty of Pharmacy, Mazandaran University of Medical Sciences, Sari, Iran; 5 Pharmaceutics Research Laboratory, School of Life Sciences, University of Sussex, Brighton, BN1 9QJ UK

**Keywords:** Cumin, Cuminum cyminum, Diclofenac, Essential oil, Nanoemulsion, Permeation enhancers

## Abstract

Essential oils have been proposed as promising non-toxic transdermal permeation enhancers. Their use is limited because of their low water solubility. The use of nanotechnology-based strategies is one of the ways to overcome this limitation. This study aimed to explore the transdermal permeation enhancing capability of cumin essential oil in nanoemulgel systems containing diclofenac sodium. Cumin essential oil nanoemulsion was produced by high-pressure homogenization technique. The formulation was optimized by changing HLB values in a range of 9.65–16.7 using different surfactant mixtures, namely, Tween 20, Tween 80, and Span 80. Preparations were characterized by polydispersity index, droplet size, and zeta potential. Nanoemulsion with concentrations of 2 and 4% essential oil was incorporated into 0.75% Carbopol gel matrix to make nanoemulgel formulation, and its permeation enhancing effect was performed through Franz diffusion cells. Antinociceptive activities of the formulations were measured in thermal (tail-flick) and chemical (formalin) models of nociception in mice. Characterization exhibited that at HLB value of 9.65, the smallest particle size (82.20 ± 5.82 nm) was formed. By increasing the essential oil percentage in the nanoemulgel from 1 to 2%, the permeation of diclofenac increased from 28.39 ± 1.23 to 34.75 ± 1.07 µg/cm^2^ at 24 h. The value of permeation from the simple gel (21.18 ± 2.51 µg/cm^2^) and the marketed product (22.97 ± 1.92 µg/cm^2^) was lower than the formulations containing essential oil. Nanoemulgel of diclofenac containing essential oil showed stronger antinociceptive effects in formalin and tail-flick tests than simple diclofenac gel and marketed formulation. In conclusion, the study proved that nanoemulgel formulation containing cumin essential oil could be considered as a promising skin enhancer to enhance the therapeutic effect of drugs.

**Graphical abstract:**

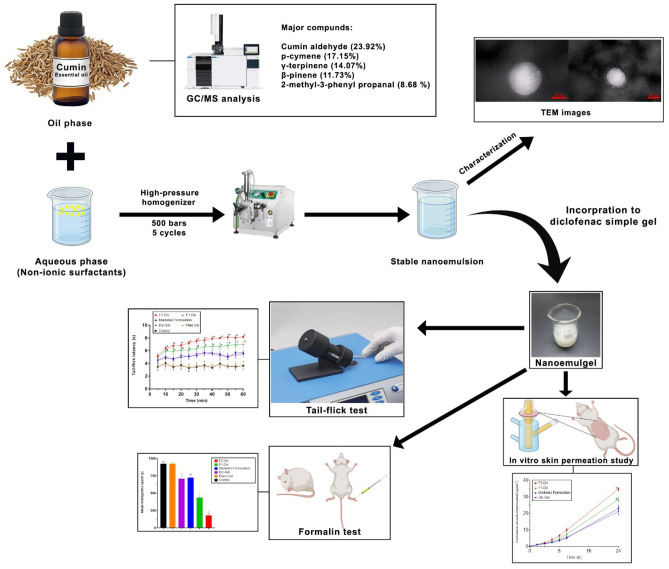

**Supplementary information:**

The online version contains supplementary material available at 10.1007/s13346-021-01025-1.

## Introduction

Topical drug administration may offer several advantages over oral delivery such as avoiding the first-pass metabolism, enhancing patient acceptance (i.e., non-invasiveness), immediate treatment withdrawal, and continued medication supply to provide constant plasma profiles, particularly for drugs with a short biological half-live [1, 2].

Topical formulations should be simple and acceptable to use, and they should mainly penetrate the skin and permeate sufficiently to the skin to exercise a therapeutic effect into the target areas [1]. Despite the significant potential of these products for dermal application, a very small number of formulations are available commercially because of the stratum corneum barrier function, which limits the permeation of most exogenous substances [2, 3], and it is not an easy task to overcome the poor permeability of the stratum corneum.

One of the most popular ways of improving the performance of transdermal formulation is using permeation enhancers in the formulation to increase the permeability of the stratum corneum temporarily and reversibly for a while. The increase in the permeability of the stratum corneum provides safe and efficient delivery of medicines via the skin [4]. Different classes of chemicals, e.g., alcohols, azones, chelating agents, essential oils, and their constituents, pyrrolidones, sulphoxides, and surfactants, have been explored and tested over the years [5].

Essential oils are complex combinations of terpene/terpenoids, phenylpropanoids, and small quantities of miscellaneous volatile organic materials [4].

The composition of essential oil could vary based on geographical and climatic conditions, as well as harvest time. Consequently, the plant’s adaptive metabolism affects the content, amount, and chemical properties of the essential oil that have an impact on the therapeutic characteristics of the essential oil extracted from the plant [6]. Certain essential oils and terpenes were proposed as promising drug penetration boosters for transdermal delivery of drugs [5, 7–9]. Unfortunately, essential oils are volatile and are easily decomposed by light, heat, humidity, and oxygen, which can reduce their effectiveness as skin enhancers [10].

One of the new unique ways for drug delivery is nanolipoidal systems. Among the different nanolipoidal delivery strategies (e.g., liposomes, solid lipid nanoparticles, nanostructured lipid carriers, microemulsion, and nanoemulsion), nanoemulsions are one of the effective delivery strategies for lipophilic active agents [11]. The nanoemulsion is a nano-vehicle that has a high performance for transdermal applications and can incorporate numerous lipophilic constituents, efficiently providing lipophilic substances on the skin whilst protecting the constituent components [10, 12].

Nanoemulgel is a nanoemulsion that contains a gelling ingredient [13]. According to various experimental findings regarding emulgel dosage forms, a drug from nanoemulgel can permeate the skin via both transcellular and paracellular routes, whereas nanoemulsion only delivers the drug via the transcellular route [14, 15].

Cumin (*Cuminum cyminum* L.), an annual herbaceous plant of the Apiaceae family, grows to approximately 25 cm in height. Cumin is originally native to Central Asia, Egypt, and the East Mediterranean region, but it is extensively planted in Algeria, China, India, Indonesia, Iran, Japan, Morocco, Turkey, and Southern Russia [16]. In conventional medicine, cumin has been used extensively to treat flatulence, digestive diseases, diarrhea, and wounds [17]. Recently, exploring the chemical composition of cumin seed (rich in essential oils) has attracted the attention of researchers towards the application of cumin essential oils. The main reason for the attraction was the presence of oxygenated monoterpenes or monoterpene hydrocarbons in the cumin essential oils [18]. It has been shown that terpenes are one of the safest and effective classes of chemical penetration enhancers and are classified as generally regarded as safe (GRAS) by Food and Drug Administration [19]. Therefore, it is expected to have a permeation enhancing effect by cumin essential oil when it is incorporated in the formulation for topical use.

Diclofenac sodium is a potent nonsteroidal anti-inflammatory drug (NSAID) that is widely used in musculoskeletal and inflammatory disorders. It is a non-specific cyclooxygenase inhibitor (Cox-1 and Cox-2) inclined to bind to some prostaglandin receptors and causes serious side effects such as stomach bleeding, ulceration, and perforation of the intestinal wall [2]. Due to first-pass hepatic metabolism, approximately 50% of the dose is available biologically, which led to a short biological half-life (~ 2 h) when is given orally [20]. Because of its gastrointestinal side effects, short biological half-life, and low bioavailability, alternative route such as transdermal delivery is preferred over oral delivery. As the skin absorption of diclofenac sodium is poor, therefore, the current study aims to enhance its skin absorption as a new nanoemulgel formulation containing cumin essential oil (CE) as a skin enhancer. Furthermore, this study promotes the further application of CE in combination with nanoemulgel formulations.

## Materials and methods

### Materials

Carbopol 940 was kindly supplied by BF Goodrich Co (UK). Triethanolamine and formalin were purchased from Sigma-Aldrich (St Louis, MO). Diclofenac sodium was kindly provided by Sohban Pharmaceutical Co (Tehran, Iran). Tween 20, Tween 80, and Span 80 were purchased from Merck (Darmstadt, Germany). Barij Essence Pharmaceutical Company kindly provided the *Cuminum cyminum* seed essential oil collected from Kerman Province in Iran. Deionized water was purified by Human Power II + Scholar (Human Corporation, Korea). Methanol (HPLC grade) was purchased from Merck (Darmstadt, Germany). Acetic acid was supplied by Beijing Chemical Reagent Co (China). All utilized chemicals were of analytical grade.

### Essential oil analysis

The chemical composition of cumin essential oil (CE) was analyzed in a fused-silica (30 m × 0.25 mm, film thickness 0.25 μm) capillary DB-5 gas chromatography (GC) interfacing with a 5975C mass spectrometer (Agilent Technologies, Palo Alto, CA). The carrier gas was helium with a 1-mL/min flow rate. Essential oil (1 μL) was injected into the GC by split mode with a split ratio of 1:100. The detector and injector temperature were maintained at 250 °C. The oven temperature was programmed to be heated at 50 °C for 5 min and raised to 200 °C at a rate of 5 °C/min. The temperature was then maintained for 5 min and ramped back to 280 °C at a rate of 10 °C/min and held for 2 min. The components were identified by comparing their mass spectra with multiple mass spectral libraries (including NIST08 and Wiley7n.l) and by comparison of their retention index calculated against n-alkanes [21]. The percentage of the compounds was calculated from the GC peak area without a correction factor.

### Formulation of nanoemulsions of CE

The coarse emulsion was prepared by dispersing CE into the deionized water and Tween 80, Tween 20, or a mixture of Tween 80/Span 80 as emulsifiers. The concentration of surfactant(s) was kept constant at 3 wt%. A range of HLB values from 9.65 to 16.7 was utilized to prepare CE nanoemulsion, and the influence of HLB value was investigated (Table 1). The mixture was then homogenized by a high-speed homogenizer (Silent Crusher M, Heidolph, Germany) at 8000 rpm for 10 min. The obtained coarse emulsions were then subjected to homogenization by utilizing an FBF laboratory high-pressure homogenizer (Italy) at 500 bars for five cycles. The device was covered with ice bags to prevent an increase in the temperature. Optimum conditions have been selected for further investigation based on droplet size and particle size distribution.

**Table 1:** Composition (wt%), mean droplet size (nm), polydispersity index (PDI), and zeta potential (mV) of CE nanoemulsion

Nanoemulsion Code	CE (%)	Tween 80 (%)	Span 80 (%)	Tween 20 (%)	Water (%)	HLB	Size	PDI	Zeta
NE1	1	-	-	3.0	96	16.70	155.50 ± 4.81	0.28 ± 0.05	− 2.24 ± 0.37
NE2	1	3.0	-	-	96	15.00	136.65 ± 15.06	0.39 ± 0.01	− 2.67 ± 0.24
NE3	1	2.0	1.0	-	96	11.43	114.65 ± 11.95	0.27 ± 0.01	− 4.63 ± 1.59
NE4	1	1.5	1.5	-	96	9.65	91.15 ± 4.06	0.26 ± 0.01	− 6.90 ± 0.81
NE5	2	1.5	1.5	-	95	9.65	87.53 ± 3.34	0.24 ± 0.03	− 3.04 ± 1.11
NE6	4	1.5	1.5	-	93	9.65	82.20 ± 5.82	0.20 ± 0.01	− 0.50 ± 0.40

The values were expressed as mean ± SD (n = 3)

### Characterization of nanoemulsions

#### Determination of particle size, polydispersity index (PDI), and zeta potential

The dynamic light scattering (DLS) with Zetasizer Nano ZS90 (Malvern Instruments, Malvern, UK) equipped with disposable capillary cuvette (DTS 1060) was used to determine the size, zeta potential, and polydispersity index (PDI) of nanoemulsion formulations. The DLS technology provides a PDI and an intensity weighted mean diameter (Z-average). The measurements were conducted at a fixed scattering angle of 90° at 25 °C. The reported values are the mean ± standard deviation (SD) of at least three determinations. The Malvern Zetasizer was also employed to measure the zeta potential through electrophoretic mobility measurements. The zeta potential was measured using the same cuvette. The average value was calculated immediately after the measurement of particle size.

#### Morphology and shape analysis by transmission electron microscopy

The surface morphology of the prepared nanoemulsions has been determined using TEM. Briefly, one drop of nanoemulsion formulation was placed on a copper grid and stained negatively with 2 percent phosphotungstic acid. Sample air drying was carried out while any remaining solution traces were cleared with filter paper [22]. The test was observed with a 100 kV tungsten source with Philips EM 208S (Netherlands).

### Preparation of Carbopol 940 plain gel base and Diclofenac simple gel

In order to prepare a plain gel base, first, Carbopol 940 polymer (0.75 wt%) was dispersed in distilled water and was allowed to swell in the dark at 25 ± 2 °C for 24 h to remove air bubbles and allow to reach the equilibrium condition. Finally, the pH value of the polymeric solution was adjusted to 6 using triethanolamine after the swelling of the polymer and result in a transparent and clear gel.

Formulation of diclofenac simple gel (Dic-Gel) was prepared by the addition of diclofenac sodium (1 wt%) in distilled water containing 0.75% Carbopol. The addition of the drug was performed under stirring conditions. The steps were completed as mentioned for the Carbopol 940 plain gel base.

### Preparation of nanoemulgel

All nanoemulsion formulations were found in the nano-size range and thus included in the gel matrix resulting in nanoemulgel. The nanoemulgels were prepared by mixing nanoemulsion into Dic-Gel containing Carbopol 940. First, the Dic-Gel was prepared as described earlier. Then, the Dic-Gel was mixed separately with nanoemulsion at a 1:1 (wt%) ratio and was stirred (120 rpm) at room temperature until the nanoemulgels were clear and homogeneous. Based on Table 1, preliminary screening formulations with different HLB values (NE1–NE4) were prepared. Based on these 4 formulations, the formulations that were optimized at the optimum HLB with higher concentrations of essential oil (listed as NE5 and NE6 in Table 1) were manufactured. The gel matrix containing NE5, NE6 plus diclofenac will be referred to hereafter as F1-Dic and F2-Dic, respectively.

### HPLC methodology

Diclofenac concentrations were determined using an HPLC (Knauer, Berlin, Germany) at a wavelength of 276 nm. The solvent system contained methanol:distilled water:acetic acid (80:20:0.5; v/v/v), and Eurospher 100–5 C18 5 μm, 4.6/250 mm was used as a column (Knauer, Berlin, Germany). As described above, the standard HPLC method was used for diclofenac [23]. The injection volume was 20 μL, and the flow rate of the mobile phase was set up at 0.8 mL/min. A linear correlation was achieved between peak area and diclofenac concentrations (r^2^ = 0.9998) in the range of 1–20 μg/mL.

### Animals

In this study, 20–28 g male NMRI mice and 220–260 g male Wistar rats were used. They had the rodent’s chow-fed ad libitum and had free access to drinking water, except during the experiments. The animals were kept in a controlled room, LD 12:12 (6:00–18:00 h), and the temperature was maintained at 22 ± 3 °C. The Ethics Committee of the Mazandaran University of Medicine approved this research (approval number: IR.MAZUMS.REC.1398.1383).

### In vitro skin permeation study

The in vitro permeation study of different gel formulations containing diclofenac was compared with the marketed formulation (diclofenac 1% w/w Topical Gel, Cipla Ltd., Mumbai, India) for permeation characteristics. Cumulative diclofenac sodium permeated through the skin (µg/cm^2^) was plotted as a function of time (h) for each formulation. The slope of the graph’s linear portion was used to calculate the rate of drug penetration (flux) at steady-state.

The abdominal skin of male Wistar rats was shaved and surgically excised. The skin was carefully cleaned from respective subcutaneous fats and contacted with a saline solution 24 h before starting the diffusion experiment. For permeation studies, the system was used with three improved Franz diffusion cells. The rat skin was excised, and the stratum corneum faced the donor section and the dermis faced the receiver section. In the receiver compartment, 33 mL of deionized water was used. The diffusion cells were kept at 32 ± 0.5 °C. The solution in the receptor phase was stirred (300 rpm) during the experiment. To perform the test, 1 g of a gel formulation (listed in Table 2) was evenly dispersed in the donor region via spatula on the rasped dorsal surfaces. At different time intervals (2, 4, 6, 8, 10, and 24 h), 5-mL samples were withdrawn from the receptor phase. The equivalent volume of fresh deionized water was replaced after each sampling time. All samples were filtered using a 0.22-μm filter and analyzed using the previously described HPLC method. After the permeation study, the skin was removed and washed three times with distilled water to measure the quantity of diclofenac deposited in the skin. The skin was minced, transferred to the tube, digested in deionized water for 24 h, and then sonicated with a bath sonicator for 1 h. Then, a 0.22-μm membrane filter was used to filter the supernatant and quantified with HPLC for diclofenac content. The same experiment was carried out for the commercial product on the market (known as diclofenac sodium topical gel).

**Table 2:** Composition (wt%) of investigated formulations

Formulation code	NE5	NE6	Diclofenac	Carbopol 940	Water (qs to)
F1-Dic	50	-	1	0.75	100
F2-Dic	-	50	1	0.75	100
Dic-Gel	-	-	1	0.75	100
Plain-Gel	-	-	-	0.75	100

### Tail-flick test

The tail-flick test was performed with minor modifications in accordance with the method described elsewhere [24]. In short, 0.5 g of the product or formulation (listed in Table 2) was rubbed slowly and softly 50 times with the index finger on the mice tail (n = 6). Blank groups only received the gel base (plain-Gel without any drug). As a reference, diclofenac gel (Dic-Gel) was applied in the same way. Five minutes later, a tail-flick device (DS20, Hugo Basile, Varese, Italy) was put on the photo element window. The infrared beam is focused on the tail area, 2 cm from the base of the tail. There was a time latency recorded to react to pain stimuli. The maximum exposure to pain stimuli was limited to 10 s to prevent tissue damage. After a topical procedure, the tail-flick response was tested every 5 to 60 min.

### Formalin test

A certain amount of each formulation (0.2 g), as listed in Table 2 was applied to the dorsal surface of the left hind paw of mice (n = 6). The formulation was rubbed gently 50 times with the index finger. Blank groups only received the gel base (plain gel). Diclofenac topical gel (Dic-Gel) is applied in the same way as a reference. Five minutes later, the activity was determined through the Dubuisson and Dennis formalin test antinociceptive activity [25]. The dorsal surface of the left hind paw formalin (2.5%, 50 μL) was injected. The mice were observed for 60 min after formalin was injected and the time spent by the mice to lick the hind paw was taken as the licking time. The first 5 min post formalin injection is referred to as the early phase, and the period between 15 and 60 min is considered as the late phase [7].

### Statistical analysis

All results are indicated as mean ± SD. The significance of the data was analyzed using the analysis of variance (ANOVA) test (GraphPad Prism 8, USA) followed by multi comparison test (Tukey’s test). The *P* < 0.05 was considered significant.

## Results and discussion

### Essential oil analysis

As illustrated in Table 3, thirty-seven compounds were identified in the CE that make up 98.80% of the total composition. Based on the results of the GC–MS analysis, the essential oil was contained a high amount of oxygenated monoterpenes and monoterpene hydrocarbons. Cumin aldehyde (23.92%), *p*-cymene (17.15%), γ-terpinene (14.07%), β-pinene (11.73%), and 2-methyl-3-phenyl propanal (8.68%) were the major compounds of the CE.

**Table 3:** Chemical composition of the *Cuminum cyminum* L. essential oil

No.	Compounds	RI	GC area (%)
1	α-Thujene	924	0.39
2	Isopropylidenecyclohexane	927	0.20
3	α-Pinene	932	1.44
4	β-Pinene	974	11.73
5	Myrcene	988	0.51
6	α-Phellandrene	1002	0.36
7	α-Terpinene	1014	0.20
8	p-Cymene	1020	17.15
9	Limonene	1024	0.77
10	γ-Terpinene	1054	14.07
11	p-Cymenene	1089	1.25
12	1,3,8-p-Menthatriene	1108	0.13
13	Terpinen-4-ol	1174	0.37
14	α-Terpineol	1186	0.23
15	trans-4-Caranone	1196	0.63
16	trans-Dihydrocarvone	1200	0.33
17	Cumin aldehyde	1234	23.92
18	3-Isopropylbenzaldehyde	1236	3.64
19	2-Methyl-3-phenyl propanal	1239	8.68
20	Carvotanacetone	1244	0.30
21	Phellandral	1273	0.42
22	α-Terpinen-7-al	1283	4.62
23	γ-Terpinen-7-al	1290	1.23
24	Carvacrol	1298	0.24
25	Daucene	1380	0.44
26	β-Caryophyllene	1417	0.19
27	α-trans-Bergamotene	1432	0.16
28	Cuminic acid	1440	0.44
29	trans-β-Farnesene	1454	0.25
30	10-epi-β-Acoradiene	1474	2.22
31	ar-Curcumene	1479	0.13
32	Isodaucene	1500	0.11
33	β-Bisabolene	1505	0.14
34	α-Alaskene	1512	0.34
35	Carotol	1594	0.15
36	Dill apiole	1620	0.94
37	Isopropyl tetradecanoate	1828	0.48
Total			98.80

*RI* retention indices on a DB-5 column

Previous studies on the chemical composition of the *Cuminum cyminum* L. essential oil growing in Iran were summarized in Table S1. γ-terpinene, β-pinene, *p*-cymene, and cumin aldehyde were found to be one of the most common compounds. The results of this study were in agreement with previous studies (refer to supplementary material for more details). Differences in the composition of essential oil were known to be dependent on the pH of the soil, cultivar type, geographical origin, extraction method, harvest time, drying conditions, and storage [26].

### Droplet size and polydispersity index

The CE nanoemulsion droplet size distribution was illustrated in Table 1. Having a suitable HLB value used in the formulation was one of the major driving forces for stable nanoemulsions [27]. In the present study, the range of droplet size was from 82.20 ± 5.82 to 155.50 ± 4.81 nm. This size range with a narrow distribution caused by the high-pressure homogenization, leading to nanoemulsions with small droplet size that met the nanoemulsion criteria (less than 200 nm). Nanoemulsions with different concentrations of CE (1%, 2%, and 4% wt) showed droplet sizes of 91.15, 87.53, and 82.20 nm, respectively. A mixture of surfactants with the final HLB of 16.7 formed the biggest droplet nanoemulsion size (155.50 ± 4.81 nm). The narrow distribution with the smallest droplets can be obtained if the HLB value of the emulsifier is closer to the HLB of the oil [28]. A mixture of surfactants with the final HLB of 9.65 produces the smallest nanoemulsion droplet size. The results showed that a blend of two non-ionic surfactants with high HLB differences could produce the smallest droplet size with narrow distribution. This could be due to the presence of one surfactant with low HLB (dissolving in the oil phase) and one with high HLB (dissolving in the water) that allow these two surfactants to work well together to have a greater effect than surfactant mixtures where their HLB values were closer to each other [27, 29, 30]. Furthermore, small-sized nanoemulsion had a greater surface area. Therefore, a higher concentration of Span 80 (lipophilic) was required in the surfactant mixture to complete the surface coverage of nanoemulsion droplets in order to stabilize CE nanoemulsion [28].

The PDI is a major predictor of homogeneity and generally is utilized to determine nanoemulsion stability [31, 32]. Since the small droplet leads to a greater driving force, Ostwald ripening is one of the primary instability mechanisms in nanoemulsions [33]. The PDI has a big impact on the rate of Ostwald ripening [34]. If the PDI is < 0.08, it indicates that monodispersed value > 0.7 is an indication of wider distribution of the droplets [35, 36]. PDI value between 0.08 and 0.70 is an indication of acceptable size distribution. PDI results were represented in Table 1 showed that all formulations exhibited PDI values in the range of 0.20 ± 0.01 (NE6) and 0.39 ± 0.01 (NE2). A similar trend with particle size was found when the PDI was compared to various HLB values (Table 1). It was found that the smallest PDI (0.2) was obtained at an HLB value equal to 9.65. Nirmal et al. have performed an extensive analysis to determine the appropriate HLB for achieving anise myrtle and lemon myrtle essential oils-based nanoemulsions. The researchers have prepared nanoemulsions with different binary mixtures of emulsifiers to achieve various HLB values. They showed that the smallest droplet size and PDI were obtained for anise myrtle and lemon myrtle essential oils nanoemulsions when HLB value was 12 and 14, respectively [37]. Enayatifard et al. have prepared nanoemulsion of oregano essential oil with different ratios of Span 80, Tween 60, and Tween 20, and they showed that the lowest particle size and PDI were obtained with Tween 80 as a surfactant [22]. The different composition, structure, and interfacial layer charge covering oil droplets could be influenced by the type of emulsifier used to prepare the nanoemulsion (different HLB value).

### Particle surface charge (zeta potential)

As in nanoemulsion formulations, non-ionic surfactants were used; therefore, it was expected that these surfactants should not carry any charge. Earlier research has shown that emulsions stabilized by non-ionic surfactants may be charged because of the existence of ionizable surface-active residues (for example free fatty acids) or selective adsorption of ionic species at high pH or low pH to the droplet surfaces [38]. Another reason for this phenomenon could be the connection between the Tween 80 oxyethylene group and water molecules in the presence of hydroxyl ion at the water–oil interface [39]. The zeta potential of the nanoemulsion droplets varied from − 0.50 ± 0.40 mV (NE6) to − 6.90 ± 0.81 mV (NE4). Previous research has shown that the non-ionic emulsifiers, which create strong repulsive (steric) forces, can prevent the aggregation of the droplets because of the relatively large hydrophilic (polyoxyethylene) head groups of the Tween [38, 40].

### Morphology and TEM observation

**Fig. 1:**
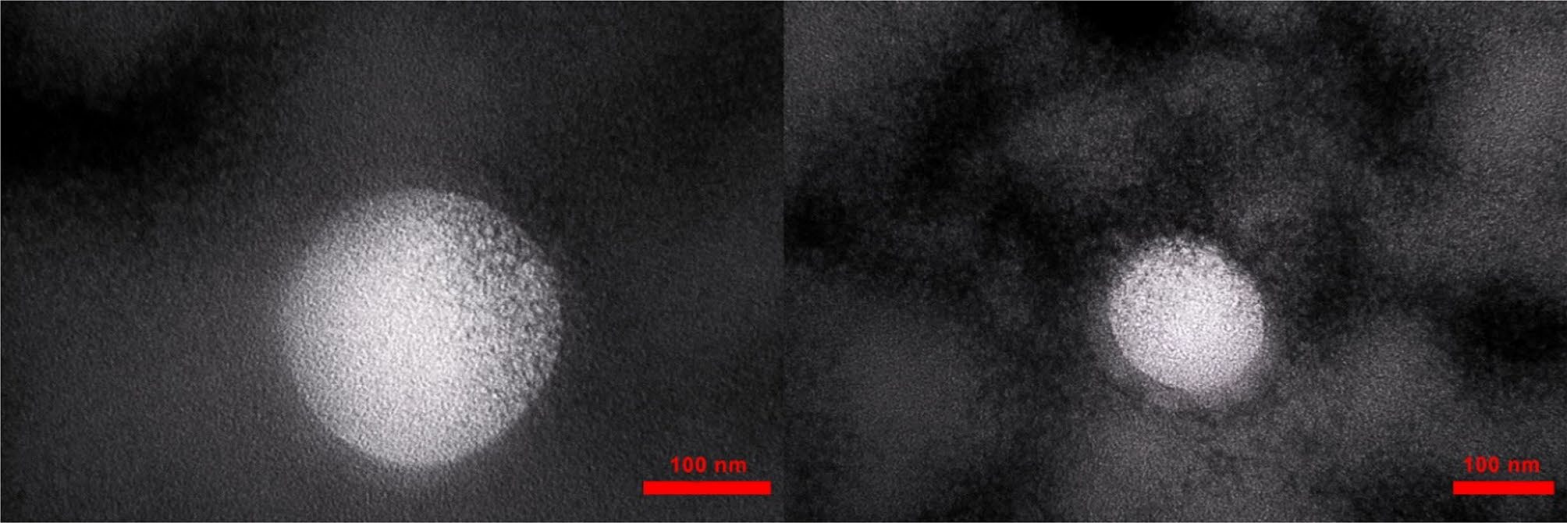
TEM images of CE loaded nanoemulsion (Formula NE5). The scale bars represent 100 nm

In the emulsion system, the morphology of droplets is an important feature [32]. Morphology of CE nanoemulsion was compiled with mixed surfactant with HLB of 9.65 at a surfactant-to-oil ratio of 1.5. TEM images with negatively stained samples were shown in Fig. 1. The nanoemulsion droplet shape was spherical with a size around 100 nm. TEM micrograph also confirmed the results obtained from droplet size analysis using the DLS.

### In vitro skin permeation study

**Fig. 2:**
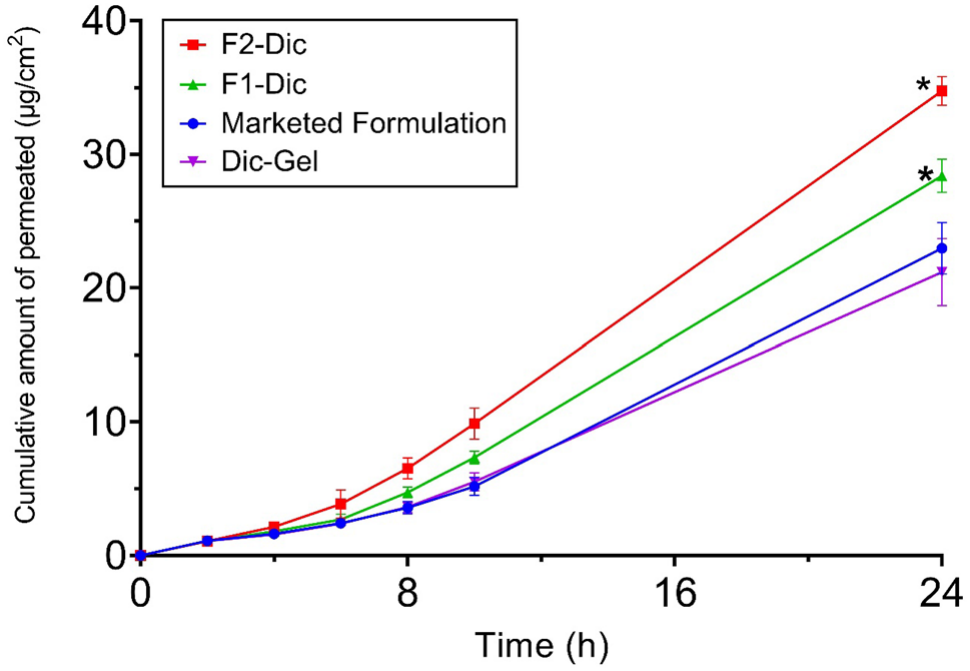
Permeation profile of diclofenac from the investigated formulation across rat skin. Data were presented as the mean ± SD of three rats. *p < 0.0001 when compared with Dic-Gel

**Fig. 3:**
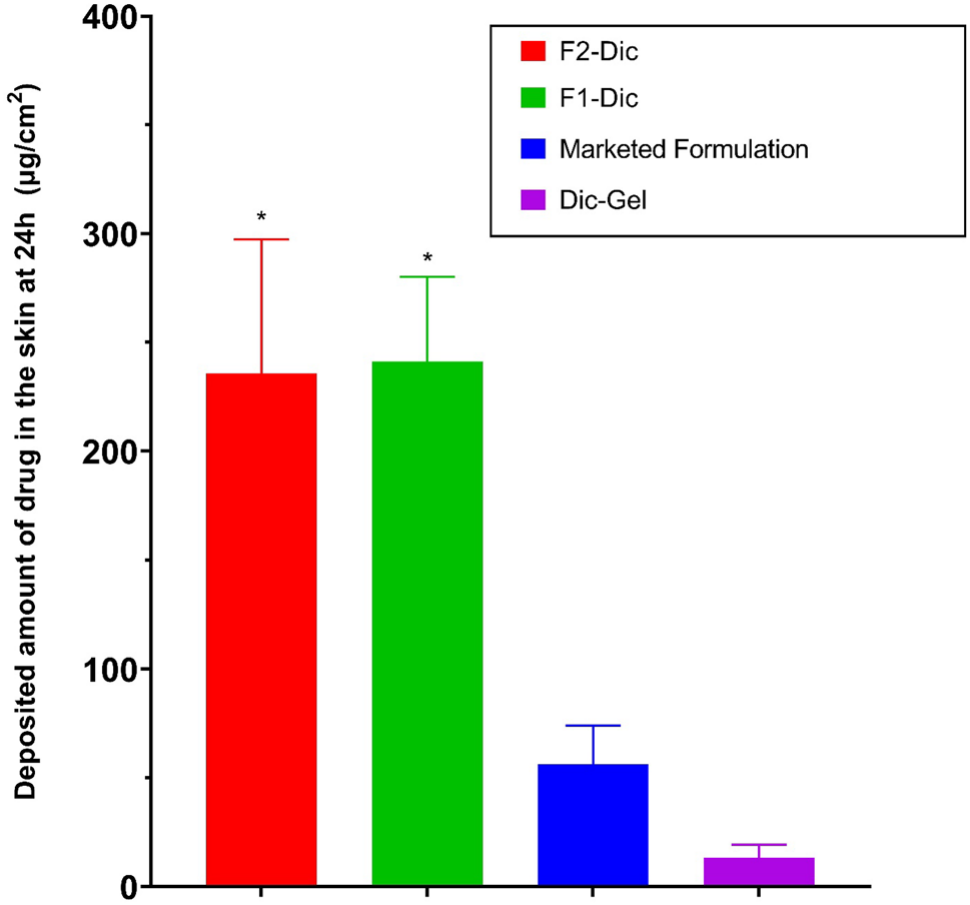
Amount of diclofenac deposited in rat skin at 24 h from the investigated formulation. Data were presented as the mean ± SD of three rats. *p < 0.001 when compared with Dic-Gel

The in vitro skin permeation studies were performed to verify the skin permeation potential of CE formulations. In particular, nanoemulgel was known to increase the permeation of drugs to deep skin layers compared to conventional formulations [41]. Rat skin was usually used as a skin model to study skin permeation [4, 42]. Figures 2 and 3 show the effects of CE nanoemulsion on the in vitro skin permeation of diclofenac (transdermal delivery) and the amount of drug permeated to the skin layers (dermal delivery), respectively. It was evident from the results that formulations containing CE showed significantly higher drug permeation than Dic-Gel and the marketed product within 24 h (*p* < 0.0001). The flux values also showed higher values for F2-Dic (1.78 ± 0.03 µg/cm^2^/h) and F1-Dic (1.50 ± 0.06 µg/cm^2^/h) compared to marketed formulation (1.27 ± 0.12 µg/cm^2^/h) and Dic-Gel formulation (1.12 ± 0.22 µg/cm^2^/h). Among the formulations, the maximum permeation value of 34.75 ± 1.07 µg/cm^2^ was obtained for F2-Dic (4% CE) followed by F1-Dic (2% CE; 28.39 ± 1.23 μg/cm^2^) at 24 h, while the permeation value of Dic-Gel (21.18 ± 2.51 μg/cm^2^) and the marketed product (22.97 ± 1.92 μg/cm^2^) was lower than the formulations contain CE nanoemulsion. The results indicated that an increase in the concentration of CE could enhance the skin absorption of the drug. The effect of CE concentration in nanoemulgel formulations on the penetration capability to deliver the drug into the skin of rats (dermal delivery) was studied (Fig. 3). The results indicated that the epidermal/dermal levels of diclofenac in F2-Dic and F1-Dic at 24 h were 235.80 ± 61.60 μg/cm^2^ and 241.29 ± 38.88 μg/cm^2^, respectively, which is 17.5 and 17.9 times higher than diclofenac simple gel (Dic-Gel). The high deposition of the drug in the skin layers indicated that the prepared nanoemulgel could provide a drug reservoir in the skin to prolong the effect of diclofenac. There was no significant difference in transdermal delivery and dermal delivery between Dic-Gel and marketed formulation.

Essential oils aided the entry of drugs into the skin by engaging in multiple physical phenomena with the lipid bilayer, such as enhanced disturbance of highly ordered intercellular lipid matrix between corneocytes, fluidization, and phase separation of the lipid portion of the stratum corneum [5]. Terpenes are important chemicals that have been used in transdermal delivery studies [43]. The enhancing effect of terpene on the skin is determined by its chemical composition and physicochemical properties, such as size, lipophilic nature, vaporization energy, boiling point, and degree of unsaturation [44]. As mentioned in the literature, relatively small terpenes, including non-polar regions, could have reinforced permeation enhancers [45]. It has also been mentioned that terpenes increase the diffusion of drugs in the skin and the distribution of drugs by affecting the lipid bilayers of the skin [46]. CE has the potential to extract lipids and cause denaturation of α-keratin that affects the composition of skin protein. It also could change the skin permeability through hydrogen bonds that influence other hydrogen bonding between the ceramides. This provides a passage in the skin for drug delivery [47, 48].

### Tail-flick test

**Fig. 4:**
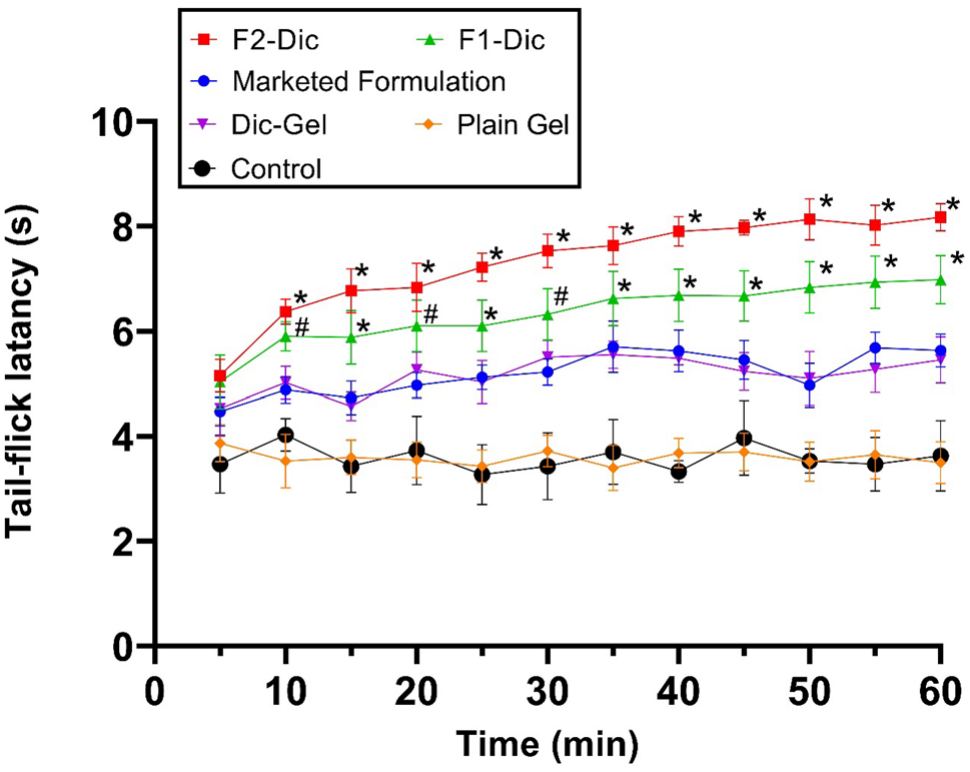
The antinociceptive effect of investigated formulation in the tail-flick test, expressed as the time-response curve. Data were presented as the mean ± SD of six mice. *p ≤ 0.0001 when compared with Dic-Gel. #*p* < 0.01 when compared with Dic-Gel

A tail-flick test is used to assess nociceptive pain and analgesic activity by measuring the time it takes to resist thermal stimuli provided by a radiant heat source [49]. Figure 4 shows the latency of tail-flick after applying the various preparations between treatment groups at each time interval. After 10 min of the topical administration, all tested formulations showed analgesic activity compared to plain gel (blank group). According to Fig. 4, the topical administration of CE nanoemulgel containing diclofenac (F1-Dic and F2-Dic) exhibited a significantly extended period of responses towards the pain stimuli in comparison to the marketed product, Dic-Gel, and plain gel (contains no drug and CE so expecting no effect) within 60 min of operation (*p* < 0.0001). The longest tail-flick latency response reported for the F2-Dic formulation was 8.17 s at 60 min after administration. These values were 6.98, 5.63, 5.45, and 3.50 s for F1-Dic, marketed formulation, Dic-gel and plain gel (blank group), respectively. The tail-flick latency time was 3.63 s for the control group. It was evident from the above findings that CE nanoemulsion in the gel system played a crucial role in improving the local analgesic of diclofenac sodium.

### Formalin test

**Fig. 5:**
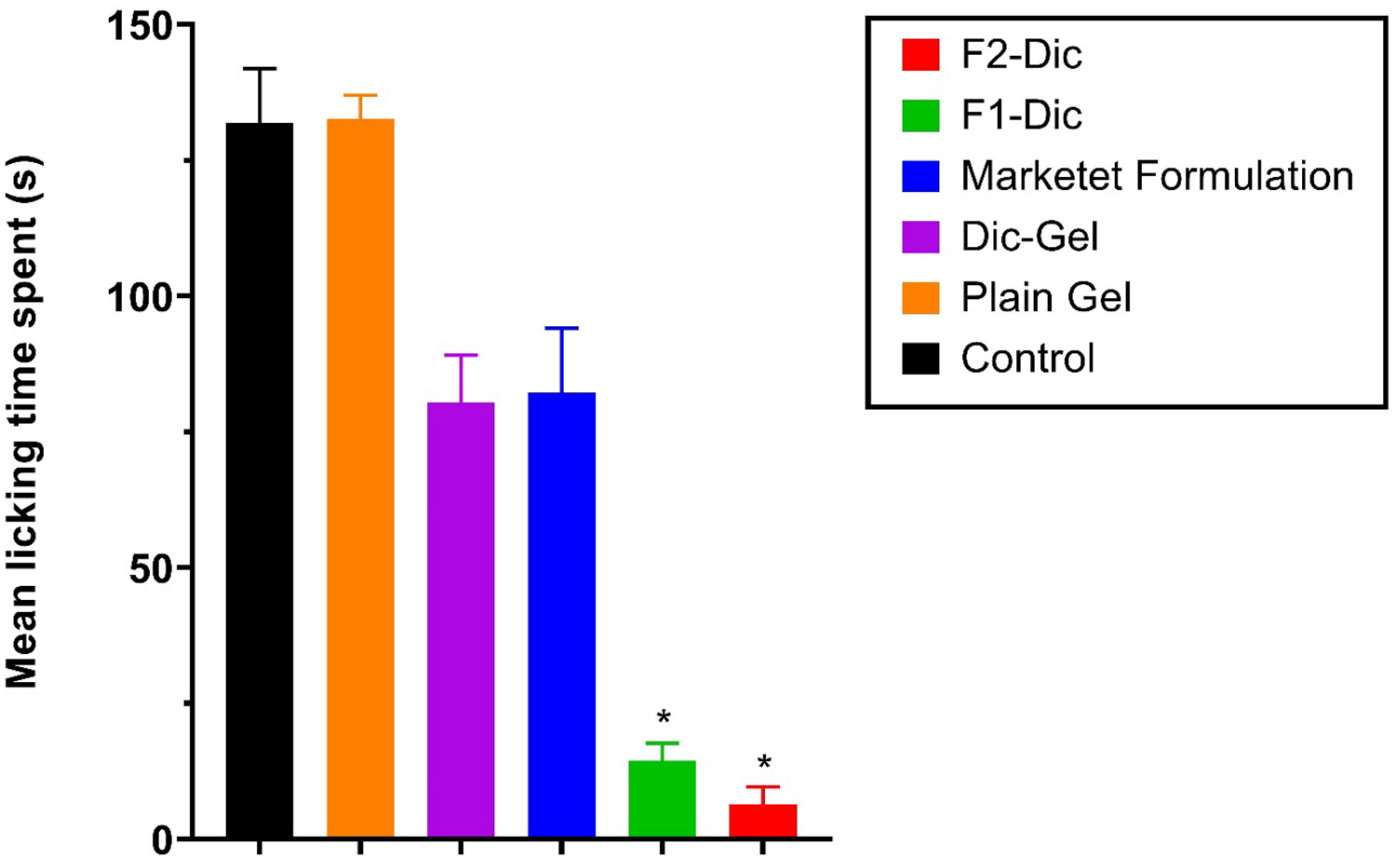
The investigated formulation’s antinociceptive effects in the formalin test on the first phase (0–5 min). Data were presented as the mean ± SD of six mice. *p < 0.0001 when compared with Dic-Gel

**Fig. 6:**
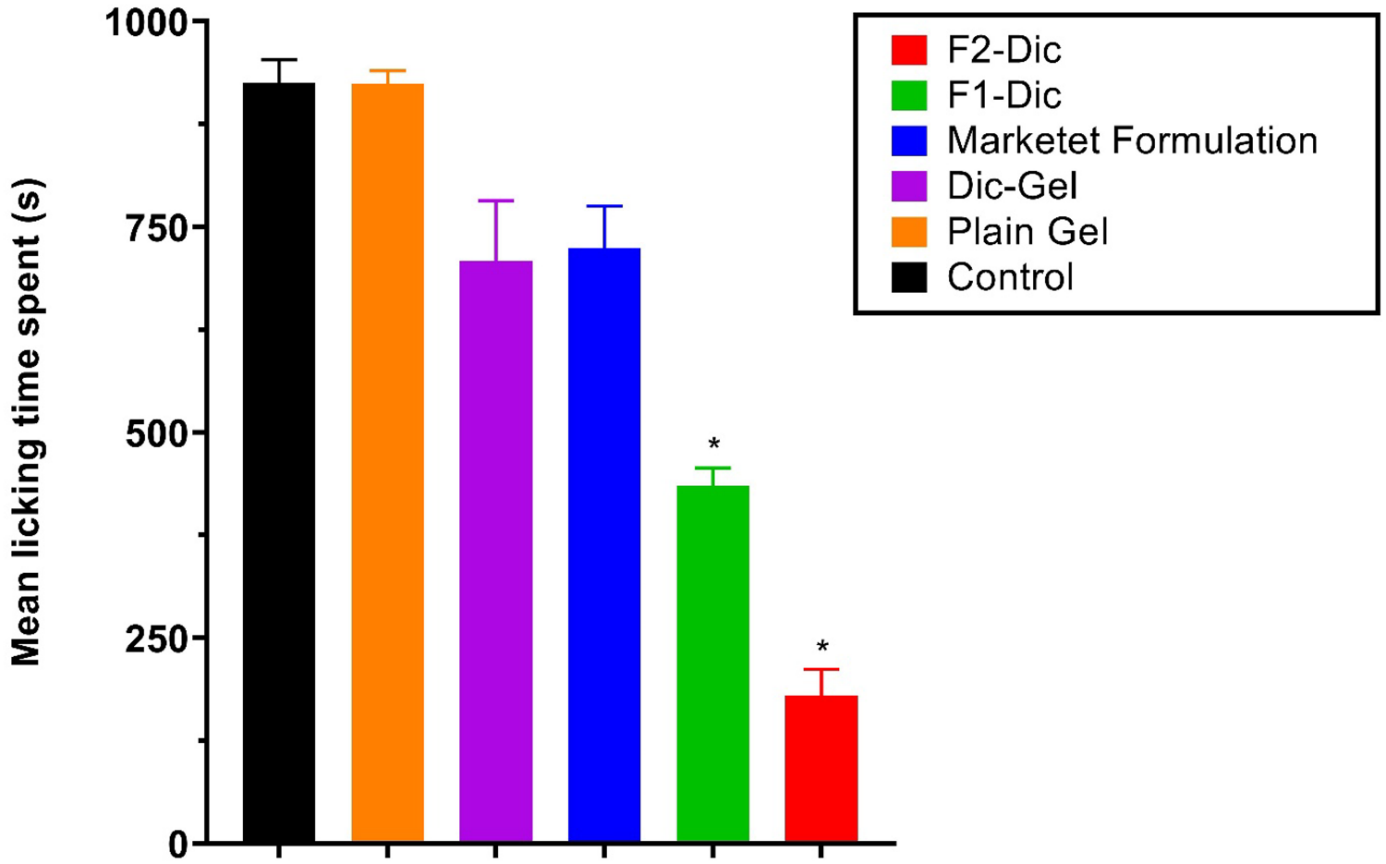
The antinociceptive effects of investigated formulation on the late-phase (15–60 min) anti-nociception in the formalin test. Data were presented as the mean ± SD of 6 mice. *p < 0.0001 when compared with Dic-Gel

Figures 5 and 6 show the formalin test results. In these figures, analgesic effects of various formulations in the early/late phases of the formalin test were demonstrated. The results (Fig. 5) suggested that the incorporation of CE nanoemulsion into the simple diclofenac gel (Dic-Gel) significantly affected the antinociceptive effect of diclofenac in the early phase. Higher analgesic effects of F2-Dic and F1-Dic were observed compared to Dic-Gel and marketed formulation in the early phase (*p* < 0.0001). The formalin test results in the late phase indicated that CE significantly affected the antinociceptive activity of diclofenac. The enhancing effects of F1-Dic and F2-Dic were observed in comparison to both Dic-Gel and marketed formulation (*p* < 0.0001) in the late phase of the formalin test (Fig. 6). No significant difference was observed in the early and late formalin test phases between the marketed product and the Dic-Gel (*p* > 0.05). The paw licking induced by the formalin test helps determine how central or peripheral pain induced is involved. There are two distinct phases in the formalin test, each reflecting different pain types. The early phase reflects the direct influence of formalin on nociceptors (pain receptor), implying non-inflammatory pain. In contrast, the late phase reflects inflammatory pain [50]. Terpenes are lipophilic compounds that can influence intercellular lipids and the non-polar penetration pathway [51]. Some terpenes are good candidates to enhance permeation due to their relatively low potential irritation [52]. This effect of essential oil improved the therapeutic effect of diclofenac sodium in nanoemulgel formulation.

## Conclusions

For the first time, this study reported the connection between diclofenac and cumin essential oil in nanoparticulate systems with its improved nociception effect in animal models. Cumin essential oil was successfully formulated as a nanoemulsion system. The produce nanoemulsion had a small droplet size and spherical morphology. The formulations demonstrated adequate physicochemical properties and enhanced diclofenac permeation through the skin of rats. The nanoemulgel formulation prolonged the tolerance of the animal against pain stimuli and formalin-induced inflammatory pain. The research demonstrates an improvement in the performance of diclofenac sodium nanoemulgel containing cumin essential oil and offers a promising avenue to enhance the penetration of NSAID drugs through the skin.

## Supplementary Information

Below is the link to the electronic supplementary material.

Supplementary file1 (DOCX 26 KB)

## Data Availability

The datasets generated during this work can be available upon request.
