# Supplementary material for: Development of a novel nanoemulgel formulation containing cumin essential oil as skin permeation enhancer
Source: Drug Deliv Transl Res. 2021 Jul 17;12(6):1455–65. doi: 10.1007/s13346-021-01025-1 (PMC9061677; doi:10.1007/s13346-021-01025-1)
Supplement: Supplementary file 1 — Supplementary file1 (DOCX 26 KB) [file 13346_2021_1025_MOESM1_ESM.docx]

Supplementary materials

Table S1. Major compounds (>5%) of the *Cuminum cyminum* L. essential oil growing in Iran

| Sample origin | Major compounds^*^ | References |
| --- | --- | --- |
| Iran | *p*-Mentha-1,4-dien-7-al (41.0%), γ-terpinene (23.2%), β-pinene (16.4%), and cumin aldehyde (13.0%) (using supercritical carbon dioxide extraction method).  *p*-Mentha-1,4-dien-7-al (27.4%), γ-terpinene (23.9%), β-pinene (16.3%), cumin aldehyde (15.7%), *p*-Mentha-1,3-dien-7-al (8.2%), and *o*-cymene (5.1%) (using steam distillation method). | Eikani *et al.* 1999 [1] |
| Iran, Sabzevar | Cumin aldehyde, and α-terpinen-7-al (using superheated water extraction method).  γ-terpinen-7-al (using hydrodistillation method).  γ-terpinen-7-al (using soxhlet extraction method). | Eikani *et al.* 2007 [2] |
| Iran, National Botanical Garden | α-Pinene (29.1%), limonene (21.5%), 1,8-cineole (17.9%), and linalool (10.4%). | Gachkar *et al.* 2007 [3] |
| Iran, Kerman Province | 2-Methyl-3-phenylpropanal (32.27%), γ-terpinene (15.82%), myrtenal (11.64%), β-pinene (6.96%), *p*-cymene (6.03%), and 2-caren-10-al (5.09%). | Jalali-Heravi et al. 2007 [4] |
| Iran, Lorestan Province | *p*-Mentha-1,3-dien-7-al (38.49%), cumin aldehyde (32.91%), γ-terpinene (8.17%), and *p*-cymene (6.08%) (using hydrodistillation method).  *p*-Mentha-1,3-dien-7-al (38.28%), γ-terpinene (21.56%), cumin aldehyde (15.89%), β-pinene (11.59%), and *p*-cymene (7.75%) (SPME method using polydimethylsiloxane ﬁber).  *p*-Mentha-1,3-dien-7-al (46.64%), cumin aldehyde (27.67%), and γ-terpinene (12.42%) (SPME method using HPTES-SBA-15 ﬁber). | Hashemi *et al.* 2009 [5] |
| Iran | α-Pinene (29.1%), limonene (21.5%), 1,8-cineole (17.9%), and linalool (10.4%). | Allahghadri *et al.* 2010 [6] |
| Iran, Tehran Province | Cumin aldehyde (25.2%), γ-terpinene (19.0%), *p*-mentha-1,4-dien-7-al (16.6%), *p*-mentha-1,3-dien-7-al (13.0%), β-pinene (10.3%), *p*-cymene (7.2%), and *p*-menth-3-en-7-al (5.1%). | Derakhshan *et al.* 2010 [7] |
| Iran, Mashhad | Cumin aldehyde (30.2%), *p*-cymene (14.1%), γ-terpinene (12.8%), safranal (9.4%), and β-pinene (6.4%). | Oroojalian *et al.* 2010 [8] |
| Iran, Alborz mountains | α-Pinene (29.2%), limonene (21.7%), 1,8-cineole (18.1%), and linalool (10.5%). | Mohammadpour *et al.* 2012 [9] |
| Iran | Cumin aldehyde (41.56%), γ-terpinene-7-al (17.14%), γ-terpinene (13.96%), α-terpinene-7-al (12.58%), and *p*-cymene (5.34%). | Morshedi *et al.* 2014 [10] |
| Iran | 1-Phenylpropanol (28.21%), γ-terpinene (23.9%), β-pinene (14.72%), cumin aldehyde (14.31%), and *p*-cymene (8.13%). | Rezai *et al.* 2014 [11] |
| Iran, Kermanshah mountains | α-Pinene (30.12%), 1,8-cineole (11.54%), linalool (10.30%), and limonene (10.11%). | Esmaeili 2015 [12] |
| Iran, Kerman Province | δ-Terpinene (22.30%), cumin aldehyde (21.09%), *o*-cymene (12.71%), β-pinene (8.46%), *p*-cymen-7-ol (6.67%), and *p*-menth-1-en-7-ol (5.99%). | Tavakoli *et al.* 2015 [13] |
| Iran | γ-Terpinene (26.24%), *p*-cymene (21.5%), cuminal (19.8%), and β-pinene (11.8%). | Habibi *et al.* 2016 [14] |
| Iran, Kurdistan Province | Thymol (40.68%), γ-terpinene (24.51%), and β-pinene (5.38%). | Ladan Moghadam 2016 [15] |
| Iran, Ilam | Thymol (40.05%), γ-terpinene (19.66%), and β-pinene (9.05%). | Ladan Moghadam 2016 [16] |
| Iran | Cumin aldehyde (29.02%), α-terpinene (20.7%), γ-terpinene (12.94%), and *p*-cymene (8.55%). | Valizadeh *et al.* 2016 [17] |
| Iran, 20 samples from different locations | γ-Terpinene (26.53-37.81%), cumin aldehyde (9.45-20.66%), cumin alcohol (1.63-15.22%), and β-pinene (8.32-13.84%). | [Moghaddam](https://www.sciencedirect.com/science/article/abs/pii/S0926669017300936#!) [and Ghasemi Pirbalouti](https://www.sciencedirect.com/science/article/abs/pii/S0926669017300936#!) 2017 [18] |
| Iran, East Azerbaijan Province, Ilkhchi | 3-Caren-10-al (47%), cuminal (25%), 2-caren-10-al (8%), γ-terpinene (%7), and (-)-β-pinene (5%). | Ghasemi *et al.* 2018 [19] |
| Iran | 1-Phenyl-1-butanol (40.8%), cuminal (28.0%) and 1-Isopropylidene-3-*n*-butyl-2-cyclobutene (9.9%). | Haddad *et al.* 2018 [20] |
| Iran, Mazandaran Province, Ramsar | γ-Terpinene (%12.57), β-pinene (%11.03), geranyl acetate (%10.81), *p*-cymene (%9.95), and sabinene (%9.81), and α-pinene (5.42%). | Nemati *et al.* 2019 [21] |

^*^Hydrodistillation is an extraction method of essential oil in many articles.

Table S2. The steady-state flux of diclofenac sodium permeation via rat skin

|  | Formulation code | | | |
| --- | --- | --- | --- | --- |
|  | **F2-Dic** | **F1-Dic** | **Marketed Formulation** | **Dic-Gel** |
| Flux (µg/cm^2^/h) | 1.78±0.03 | 1.50±0.06 | 1.27±0.12 | 1.12±0.22 |

Data were presented as the mean±SD, n=3

References

1. Eikani MH, Goodarznia I, Mirza M. Supercritical carbon dioxide extraction of cumin seeds (Cuminum cyminum L.). Flavour Fragr J. 1999;14(1):29-31. <https://doi.org/10.1002/(SICI)1099-1026(199901/02)14:1%3C29::AID-FFJ765%3E3.0.CO;2-G>.
2. Eikani MH, Golmohammad F, Mirza M, Rowshanzamir S. Extraction of volatile oil from cumin (Cuminum cyminum L.) with superheated water. J Food Process Eng. 2007;30(2):255-66. <https://doi.org/10.1111/j.1745-4530.2007.00117.x>.
3. Gachkar L, Yadegari D, Rezaei MB, Taghizadeh M, Astaneh SA, Rasooli I. Chemical and biological characteristics of Cuminum cyminum and Rosmarinus officinalis essential oils. Food Chem. 2007;102(3):898-904.
4. Jalali-Heravi M, Zekavat B, Sereshti H. Use of gas chromatography–mass spectrometry combined with resolution methods to characterize the essential oil components of Iranian cumin and caraway. J Chromatogr A. 2007;1143(1-2):215-26. <https://doi.org/10.1016/j.chroma.2007.01.042>.
5. Hashemi P, Shamizadeh M, Badiei A, Ghiasvand AR, Azizi K. Study of the essential oil composition of cumin seeds by an amino ethyl-functionalized nanoporous SPME fiber. Chromatographia. 2009;70(7-8):1147. <https://doi.org/10.1365/s10337-009-1269-7>.
6. Allahghadri T, Rasooli I, Owlia P, Nadooshan MJ, Ghazanfari T, Taghizadeh M, et al. Antimicrobial property, antioxidant capacity, and cytotoxicity of essential oil from cumin produced in Iran. J Food Sci. 2010;75(2):H54-H61. <https://doi.org/10.1111/j.1750-3841.2009.01467.x>.
7. Derakhshan S, Sattari M, Bigdeli M. Effect of cumin (Cuminum cyminum) seed essential oil on biofilm formation and plasmid Integrity of Klebsiella pneumoniae. Pharmacogn Mag. 2010;6(21):57. <https://doi.org/10.4103/0973-1296.59967>.
8. Oroojalian F, Kasra-Kermanshahi R, Azizi M, Bassami MR. Phytochemical composition of the essential oils from three Apiaceae species and their antibacterial effects on food-borne pathogens. Food Chem. 2010;120(3):765-70. <https://doi.org/10.1016/j.foodchem.2009.11.008>.
9. Mohammadpour H, Moghimipour E, Rasooli I, Fakoor MH, Astaneh SA, Moosaie SS, et al. Chemical composition and antifungal activity of Cuminum cyminum L. essential oil from Alborz mountain against Aspergillus species. Jundishapur J Nat Pharm Prod. 2012;7(2):50.
10. Morshedi D, Kesejini TS, Aliakbari F, Karami-Osboo R, Shakibaei M, Marvian AT, et al. Identification and characterization of a compound from Cuminum cyminum essential oil with antifibrilation and cytotoxic effect. Res Pharm Sci. 2014;9(6):431.
11. Rezai R, Sadeghi E, Nateghi L, Mohammadi M. The effect of Cuminum cyminum essential oil on growth and survival of Staphylococcus aureus during storage of hamburger. Int J Biosci. 2014;5(4):18-26.
12. Esmaeili F. Composition of Essential Oil of Cuminum cyminum. J Essent Oil-Bear Plants. 2015;18(2):507-9. <https://doi.org/10.1080/0972060X.2014.989186>.
13. Tavakoli HR, Mashak Z, Moradi B, Sodagari HR. Antimicrobial activities of the combined use of Cuminum cyminum L. essential oil, nisin and storage temperature against Salmonella Typhimurium and Staphylococcus aureus in vitro. Jundishapur J Microbiol. 2015;8(4). <https://doi.org/10.5812/jjm.8(4)2015.24838>.
14. Habibi R, Jalilvand G, Samadi S, Azizpour A. Effect of Different levels of essential oils of Wormwood (Artemisia absinthium) and Cumin (Cuminum cyminum) on growth performance carcass characteristics and immune system in broiler chicks. Iran J Appl Anim Sci. 2016;6(2):395-400.
15. Moghadam AL. Efficacy of chemically characterized cuminum cyminum essential oil as an antioxidant and lipid peroxidation inhibitor. J Essent Oil-Bear Plants. 2016;19(1):134-9. <https://doi.org/10.1080/0972060X.2015.1086284>.
16. Ladan Moghadam AR. Chemical Composition and Antioxidant Activity Cuminum cyminum L. Essential Oils. Int J Food Prop. 2016;19(2):438-42. <https://doi.org/10.1080/10942912.2015.1038355>.
17. Valizadeh S, Mahmodi R, Fakheri T, Katiraie F, Rahmani V. Investigating the phytochemical, antibacterial and antifungal effects of Thymus Vulgaris and Cuminum Cyminum essential oils. Med Lab J. 2016;10(1):36-43. <https://doi.org/10.18869/acadpub.mlj.10.1.36>.
18. Moghaddam M, Pirbalouti AG. Agro-morphological and phytochemical diversity of Iranian Cuminum cyminum accessions. Ind Crops Prod. 2017;99:205-13. <https://doi.org/10.1016/j.indcrop.2017.02.003>.
19. Ghasemi G, Fattahi M, Alirezalu A. A new source of oxygenated monoterpenes with phytotoxic activity: essential oil of Cuminum Cyminum L. from Iran. Nat Prod Res. 2018;34:1-4. <https://doi.org/10.1080/14786419.2018.1501686>.
20. Haddad B, Bidgolia SA, Qomic M, Asgarpanaha J. Organ toxicity and estrogen like effects of cuminum cyminum. l seed essential oil: a hormonal, histopathological and immunohistochemical study in female mice. J Pharm Sci. 2018;4:1-11. <https://doi.org/10.20431/2455-1538.0402001>.
21. Nemati V, Khomeiri M, Moayedi A, Sadeghi Mahoonak A, Sadeghi A, Yamchi A. Use of Cuminum Cyminum Essential Oil and Biarum carduchcorum Water Extract on Shelf-life Extension of lambs at Cold Storage. Nutr Food Sci Res. 2019;6(3):23-32.
